# Generative Diffusion-Based Task Incremental Learning Method for Decoding Motor Imagery EEG

**DOI:** 10.3390/brainsci15020098

**Published:** 2025-01-21

**Authors:** Yufei Yang, Mingai Li, Jianhang Liu

**Affiliations:** 1 School of Information Science and Technology, Beijing University of Technology, Beijing 100124, China; yangyf0919@emails.bjut.edu.cn (Y.Y.); ljh1070110393@emails.bjut.edu.cn (J.L.); 2 Beijing Key Laboratory of Computational Intelligence and Intelligent System, Beijing 100124, China; 3 Engineering Research Center of Digital Community, Ministry of Education, Beijing 100124, China

**Keywords:** motor imagery EEG, task incremental learning, conditional diffusion, temporal-spatial feature extraction, generative artificial intelligence

## Abstract

Background/Objectives: Motor neurorehabilitation can be realized by gradually learning diverse motor imagery (MI) tasks. EEG-based brain-computer interfaces (BCIs) provide an effective solution. Nevertheless, existing MI decoding methods cannot balance plasticity for unseen tasks and stability for old tasks. This paper proposes a generative diffusion-based task Incremental Learning (IL) method called GD-TIL. Methods: First, data augmentation is employed to increase data diversity by segmenting and recombining EEG signals. Second, to capture temporal-spatial features (TSFs) from different temporal resolutions, a multi-scale temporal-spatial feature extractor (MTSFE) is developed via integrating multiscale temporal-spatial convolutions, a dual-branch pooling operation, multiple multi-head self-attention mechanisms, and a dynamic convolutional encoder. The proposed self-supervised task generalization (SSTG) mechanism introduces a regularization constraint to guide MTSFE and unified classifier updating, which combines labels and semantic similarity between the augmentation with original views to enhance model generalizability for unseen tasks. In the IL phase, a prototype-guided generative replay module (PGGR) is used to generate old tasks’ TSFs by training a lightweight diffusion model based on the prototype and label of each task. Furthermore, the generated TSF is merged with a new TSF to fine-tune the convolutional encoder and update the classifier and PGGR. Finally, GD-TIL is evaluated on a self-collected ADL-MI dataset with two MI pairs and a public dataset with four MI tasks. Results: The continuous decoding accuracy reaches 80.20% and 81.32%, respectively. The experimental results exhibit the excellent plasticity and stability of GD-TIL, even beating the state-of-the-art IL methods. Conclusions: Our work illustrates the potential of MI-based BCI and generative AI for continuous neurorehabilitation.

## 1. Introduction

Motor neurorehabilitation aims to gradually restore activities of daily living (ADL) by promoting neuroplasticity and activating compensatory mechanisms [1]. This process typically begins with simple single-motor tasks and progressively advances to more complex multitasking training. Consequently, the rehabilitation process has a higher demand for practical and sustainable rehabilitation methods.

In recent years, rapidly developing motor imagery-based brain-computer interface (MI-BCI) technology has supported an efficient and noninvasive approach for assisting in neuroplasticity. MI is a complex neurocognitive process in which individuals internally simulate limb movements without actual physical execution. This process activates neural pathways similar to those involved in real movement, including the primary motor cortex (M1), supplementary motor area (SMA), and somatosensory regions [2]. It is characterized by event-related desynchronization (ERD) and event-related synchronization (ERS) in electroencephalographic (EEG) signals, particularly within the alpha rhythm (8–13 Hz) and beta rhythm (13–30 Hz) frequency bands, reflecting the dynamic neural changes underlying MI [3]. Related studies have revealed that the formation of motor imagery is closely related to neurophysiological features (e.g., P300 and alpha rhythms), where P300 reflects an individual’s attentional allocation to external stimuli, and alpha rhythms are associated with resting-state EEG activity and inhibition in the sensorimotor cortex [4]. Classical methods usually achieve decoding of MI tasks through specific frequency feature extraction and classification algorithms [5], but there are still significant shortcomings in adaptation and generalization to new tasks in multi-task incremental learning.

Depending on large-scale datasets and strong computer arithmetic power, DL-based artificial intelligence algorithms are widely used to automatically extract the temporal-spatial features (TSFs) of MI-EEG. Based on classical models, such as EEGNet [6], ShallowConvNet [7], and Long Short-Term Memory Network (LSTM) [8], researchers have developed various improved models. For example, Fu et al. proposed the iteratively weighted sparse group lasso (iWSGL) to optimize high-dimensional features by feature grouping and conditional entropy-based weighting [9]. Furthermore, to enhance decoding robustness, a parallel multi-scale temporal convolutional network (TCN) layer was introduced to capture detailed features [10], as well as using an end-to-end multi-branch one-dimensional convolutional network (EEG-FMCNN) to cope with low signal-to-noise ratios and inter-subject variability [11]. In addition, a Riemannian geometric and a deep domain adaptation network (RGDDANet) were combined with 1D convolution to capture temporal and spatial features, further improving MI classification performance [12]. Moreover, some advanced studies have shown that Transformer has significant advantages in capturing global dependencies [13,14]. Nevertheless, most of the existing decoding methods are underperforming and jointly trained modes; they cannot learn new data while avoiding catastrophic forgetting of acquired knowledge as the human brain does. Thus, it is still an existing challenge to develop incremental decoding methods to meet the practical needs in real-world clinical rehabilitation.

Incremental learning (IL) methods are intended to continuously acquire new knowledge while effectively retaining previous knowledge by balancing the plasticity and retainability of models [15]. Currently, IL strategies mainly include parameter isolation, regularization and replaying. Parameter isolation achieves learning by freezing the old task parameters and expanding the branching for the new task [16,17], but insufficient convergence between branches may destabilize the feature space. The regularization strategy preserves previous knowledge by introducing constraints [18]; however, it is prone to lead to task interference or suboptimal convergence when the feature distributions between tasks differ significantly. Old data replay consolidates learned tasks by utilizing historical data [19,20], but its dependence on storage capacity and data privacy limits the application scenarios.

Several researchers have recently developed IL methods to facilitate the application of EEG-based BCI technologies in practical rehabilitation scenarios. In emotion recognition, the preserved learned feature space method (IL2FS) incorporating bias correction is proposed to ensure inter-class separation and intra-class alignment between old and new tasks [21]. In chronic disease diagnosis, an incremental multimodal self-encoder was developed to provide a scalable modeling framework for new tasks [22]. For the problem of imagined speech recognition, researchers proposed class-incremental neural networks that extend the vocabulary by a novel loss function that minimizes the center distance [23]. Deng et al. designed an empirical replay-based center-of-mass matching method (ER-CM) to achieve a unified incremental learning system for epilepsy prediction [24]. In addition, a meta-autonomous attention prototype incrementer (MAPIC) was proposed for medical time-series data to enhance the class incremental learning capability under few-sample conditions [25]. In terms of decoding online sequential EEG signals, Duan et al. introduced an approach based on dynamic memory buffers and kernel topic drift detection to successfully address catastrophic forgetting and maintain high performance across sequential topics [26]. Nowadays, IL methods still face many challenges in decoding incremental MI-EEG data, including feature distribution complexity, inter-task interference, and the effectiveness of model expansion.

In recent years, generative replay-based IL methods have developed rapidly, with generative models represented by the generative adversarial network (GAN) being the most widely applied, such as deep generative replay and Introspective GAN [27]. Unlike the GAN-based generation mechanism, which relies on complex adversarial training between generators and discriminators, variable Autocoder (VAE) [28], a special probabilistic model, can generate old data by maximizing the log-likelihood estimation. Ho et al. designed a diffusion model (DM) based on U-Net, called the denoising diffusion probabilistic model (DDPM), to improve the quality of the generated images [29]. Meng et al. used DM in incremental learning to fit the feature distribution of the old task, and then the generated features were employed to update the IL model with the new task. The authors of [30] confirmed that the model generates features with lower computational cost than generating data, and the classifier obtains better classification results.

In BCI studies, DM is commonly applied to generate corresponding image information from signals. Yang et al. introduced classification features as conditions to fine-tune the pre-trained stable model so that its image generation process was semantically supervised [31]. Similarly, Qian et al. combined DM with Visual-Transformer to further improve the realism of the generated images [32]. In addition, DM-based data enhancement methods are widely used to address the lack of EEG data and to increase sample diversity, including emotion recognition [33], seizure prediction [34] and MI decoding [35]; all the proposed methods showed significant reliability in data generation. Chetkin et al. developed an unconditional EEG synthesis method for MI tasks that reduces computational consumption while maintaining the generation quality [36]. Although the DM-based method can significantly improve EEG classification results, due to the large differences in the feature distribution of EEG data across MI tasks, it still has not been effectively used in task IL scenarios.

At present, although the reported IL methods have been reported to have been effectively applied to session-incremental learning and domain(subject)-incremental learning, the significant variability between MI tasks makes task-incremental learning still a great challenge. Firstly, reported methods have not yet involved decoding incremental MI tasks, whereas training tasks in practical neurorehabilitation must progressively strengthen from a single movement to multiple movements. Secondly, existing feature extractors are insufficient for temporal-spatial feature (TSF) representation. Moreover, most DM-based old data generation models lacked proper conditions to guide the generation process, resulting in low-quality generated features.

To deal with the abovementioned problems, we propose GD-TIL, a generative diffusion-based task incremental learning method, for decoding incremental MI tasks. The main contributions of this paper are as follows:

- A novel generative diffusion-based task incremental learning method (GD-TIL) is proposed by integrating self-supervised learning and generative replay strategies to continuously decode EEG-based incremental MI tasks.
- A multiscale temporal-spatial feature extractor (MTSFE) is developed to capture temporal-spatial features (TSFs) at varying temporal resolutions while boosting generalization for unseen tasks by enhancing multi-view TSF correlations.
- To balance the plasticity and stability of the model, a prototype-guided generative replay module (PGGR) is designed to generate TSFs of old tasks, which are used to fine-tune the partially parameter-frozen MTSFE with new task TSFs and update PGGR to fit all seen tasks.
- GD-TIL is evaluated on a self-collected ADL-oriented MI dataset and a public dataset. Experimental results show that the decoding ability of GD-TIL is superior to related methods, and our work showcases the potential of IL methods for application in BCI-based neurorehabilitation.

## 2. Materials and Methods

**Figure 1:**
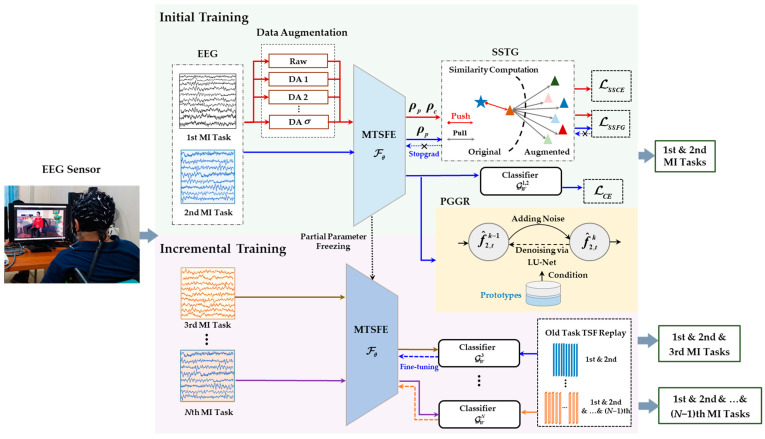
Illustration of GD-TIL, where σ represents the number of data augmentations, MTSFE represents the multiscale temporal-spatial feature extractor $F_{\theta }$, PGGR represents the prototype-guided generative replay module, SSTG represents self-supervised task generalization, TSF represents temporal-spatial feature, $\rho_{p}$ and $\rho_{c}$ are the projection header and the classifier header, respectively, and ${\hat{f}}_{2,t}^{k-1}$ and ${\hat{f}}_{2,t}^{k}$ are the added noise TSFs of the 2nd MI task in the *k* − 1 and *k* discrete time steps, respectively.

In this section, the implementation details of the proposed GD-TIL will be described; an illustration is shown in Figure 1. The model training is divided into two processes, i.e., the initial and incremental training phases. In the initial training phase, an MTSFE with strong TSF representation and MI task generalization capability and a unified classifier is developed, additionally, a PGGR is trained to prepare for the replay of old task TSFs in continuous learning. In the incremental phases, the generated old task data is merged with the new task data to fine-tune the MTSFE and update the classifier and PGGR.

### 2.1. Materials and Preprocessing

#### 2.1.1. Self-Collected ADL-Oriented MI Dataset

To advance the neurorehabilitation of stroke patients, we developed an assisted rehabilitation platform oriented to MI-BCI, called the Motor Imagery-Assisted Rehabilitation (MI-AR) platform. It implements communication with the NeuroScan SynAmps2 device for EEG signal acquisition and preprocessing and synchronizes data in real-time with a MySQL database to record and store personal information [37]. One of the MI-AR platform’s major advantages is its ability to design personalized rehabilitation programs according to the specific rehabilitation needs of stroke patients. The unique MI guidance paradigm combines auditory instructions with dynamic visual animations to simulate diverse experimental scenarios to be chosen by the participant. This design can significantly stimulate the participant to enter and actively participate in the MI task quickly, and even for individuals who are completely inexperienced in MI training, it also shows good adaptability and participation.

**Figure 2:**
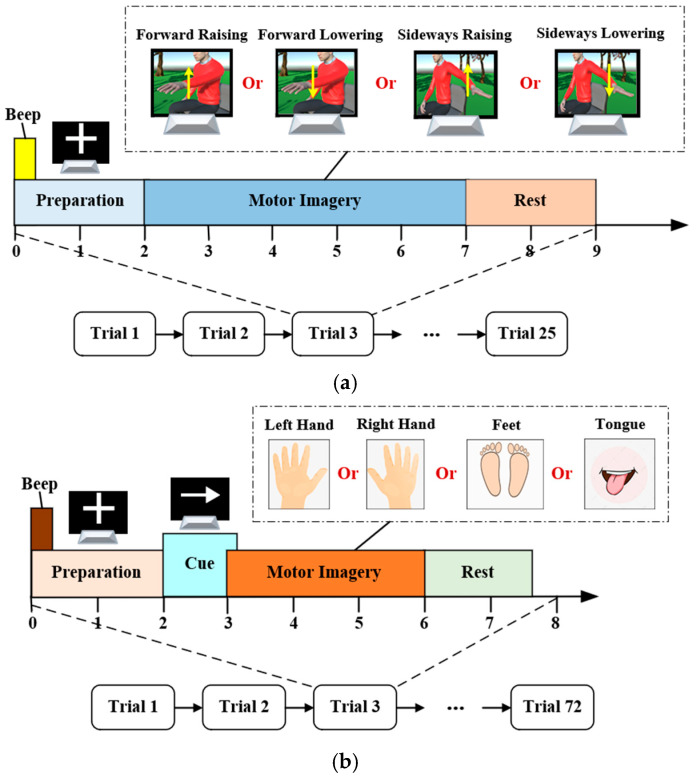
The timing scheme for the EEG acquisition process within a session. In the ADL-MI dataset, 25 sampled trials were recorded per session, with each MI task execution lasting 5 s. In the IV-2a dataset, each MI task recorded 72 sampled trials per session, with MI performed for 3 s in each trial. (**a**) ADL-MI dataset, (**b**) IV-2a dataset.

Based on the MI-AR platform, we recruited six right-handed volunteers aged 23 to 29 to conduct an EEG data acquisition experiment targeting the EEG-based decode study for incremental MI tasks. Before the experiment, all subjects had not received any MI training, and they signed a written informed consent form to ensure ethical safeguards. The positions of the 64 EEG channels followed the international 10–20 system. The sampling frequency was set to 1000 Hz. Four MI tasks were selected on the six sessions with the same time interval, including the left arm forward raising (FR) and lowering (FL), and the left arm sideways raising (SR) and lowering (SL). Each session contained 25 sampling trials, a trial lasting 18 s. The timing scheme for the EEG acquisition process within a session is shown in Figure 2a.

**Figure 3:**
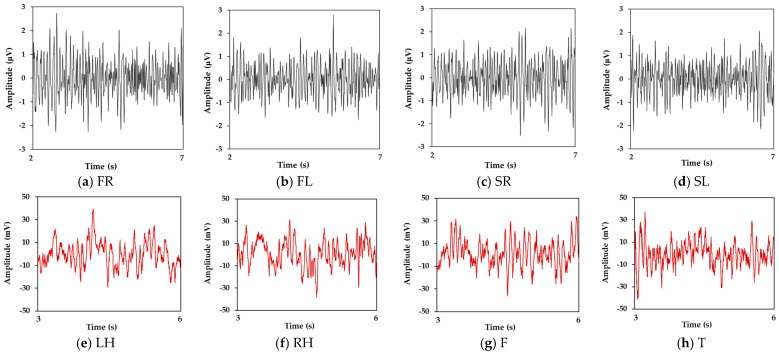
Original EEG segments. (**a**–**d**) show a trial for subject A1 from the ADL-MI dataset. (**e**–**h**) show a trial for subject B1 from the IV-2a dataset, where the amplitude is the mean value of all EEG channels. ADL-MI contains 64 channels and IV-2a contains 22 channels.

Before performing a motor imagery task, subjects were asked to sit in front of a computer and stay relaxed and stable. They were instructed to minimize blinking, avoid eye movements, and refrain from any physical movements during MI. At 0 s, a brief auditory beep served as a cue for the subjects to focus and prepare for the upcoming task. Simultaneously, a fixation cross (“+”) was displayed at the center of the screen from 0 s to 2 s to maintain their attention. From 2 s to 7 s, an animated cue depicting the MI task was presented, and subjects performed MI according to visual guidance while avoiding any actual motor execution. Following the MI phase, the screen turned blank at 7 s. Subjects were allowed a brief rest period from 7 s to 9 s before proceeding to the next sampling trials. After the EEG collection experiment, a self-collected ADL-oriented MI (ADL-MI) dataset was recorded for this study. The original EEG segments for each MI task of subject A1 are shown in Figure 3a–d, where the amplitude is the mean value of 64 EEG channels.

#### 2.1.2. BCI Competition IV-2a

The BCI Competition IV-2a (IV-2a) [38] dataset recorded EEG from nine subjects, including four MI tasks, i.e., left hand (LH), right hand (RH), feet (F) and tongue (T). Each subject completed two sessions on separate days; a session recorded 288 sampling trials. The 22 channels were arranged according to the International 10–20 system; the sampling frequency was 250 Hz. Each trial lasted 7.5 s, where MI was performed from 3 s and 6 s. The timing scheme for the EEG acquisition process within a session is depicted in Figure 2b. In particular, at 2 s to 3 s, the computer monitor provided arrow cues, the different arrow directions having been agreed upon for the corresponding tasks before the experiment. The dataset is publicly accessible at http://www.bbci.de/competition/iv/#download, accessed on 22 September 2024. The original EEG segments for each MI task of subject B1 are presented in Figure 3e–h, with the amplitude representing the average value across 22 EEG channels.

#### 2.1.3. Preprocessing

Both datasets were preprocessed via the EEGLAB toolbox [39]. First, the raw MI-EEG data were down-sampled to 250 Hz, followed by bandpass filtering within the range of 0.5–100 Hz. Then, the validity of each channel was assessed, confirming valid EEG channels 64 (ADL-MI) and 22 (BCI-IV-2a) for subsequent analysis, respectively. A global average reference was applied for re-referencing to further improve the signal quality. Next, independent component analysis (ICA) was used to remove artifacts, which were caused by electromyographic (EMG) activity, eye movements, and so on. Finally, an 8th-order Butterworth bandpass filter with a frequency range of 8–32 Hz was applied to extract the highly correlated subbands with MI. After the datasets were preprocessed, the data dimensions of each MI task ***#***trials × ***#***channels × ***#***sampling points were (25 × 6) × 64 × 1250 and (72 × 2) × 22 × 750 for ADL-MI and IV-2a datasets, respectively.

### 2.2. Problem Statement

In real-world neurorehabilitation scenarios, MI tasks are scaled incrementally from single to multiple task data chunks. The IL method aims to construct a self-updating model that sequentially adapts to new MI tasks while preserving decoding performance on all seen tasks. Specifically, the training process of the proposed method consists of an initial learning phase and subsequent *N* − 1 incremental learning phases; the EEG-based MI task set can be given as $\left\{T_{1},\ldots T_{n},\ldots ,T_{N}\right\}$. For the *n*th MI task, the data chunk is denoted as $T_{n}=\left\{X_{n},Y_{n}\right\}$, where $X_{n}\in {\mathbb{R}}^{T_{n}\times C_{n}\times V_{n}}$ is the preprocessed EEG, $T_{n}$, $C_{n}$ and $V_{n}$ represent the number of sampling trials, EEG channels and sampling points, respectively, and $Y_{n}\in {\mathbb{R}}^{T_{n}\times L_{n}}$ are the MI task labels, where $L_{n}$ is denoted as the number of cumulative labels categories, including all known categories for the first *n* tasks.

### 2.3. Details of GD-TIL

#### 2.3.1. Data Augmentation

The high decoding performance of artificial neural networks depends on the amount of training data, but EEG data acquisition is time-consuming and costly, resulting in a limited amount of training data. To this end, we enhance the data using the signal segmentation and reorganization method. Specifically, in each iteration of network training, EEG data from the same category are first divided into $s_{n}$ equal segments along the time dimension. Then, while keeping the original temporal order constant, segments from different training trials are randomly selected and exchanged to restructure augmented EEG data ${\hat{X}}_{n}=\left[{\hat{X}}_{n}^{0},{\hat{X}}_{n}^{1},\ldots ,{\hat{X}}_{n}^{\sigma }\right]$, where ${\hat{X}}_{n}\in {\mathbb{R}}^{(\sigma +1)T_{n}\times C_{n}\times V_{n}}$ and σ is defined as the number of data exchanges, and ${\hat{X}}_{n}^{0}$ are the original data unchanged. Meanwhile, the augmented EEG is relabeled as ${\hat{Y}}_{n}=\left[Y_{n}^{0},Y_{n}^{1},\ldots ,Y_{n}^{\sigma }\right]$.

#### 2.3.2. Multi-Scale Temporal-Spatial Feature Extractor

**Figure 4:**
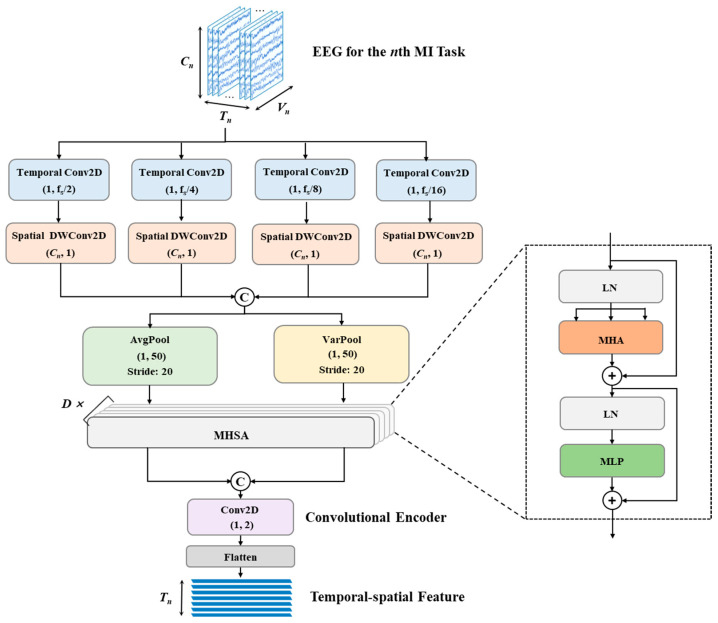
The network architecture of the multi-scale temporal-spatial feature extractor, where MHSA represents the multi-head self-attention mechanism, MHA represents multi-head attention, MLP represents multilayer perceptron, LN stands for layer normalization operation, *D* is the number of MHSA, f_s_ is the sampling frequency, and “C” symbolizes concatenating feature maps from different scales.

To capture the TSFs of high-dimensional and time-varying MI-EEG, based on convolutional operations and the multi-head self-attention mechanism (MHSA), a multi-scale temporal-spatial feature extractor (MTSFE), noted as $F_{\theta }$, is developed to extract the TSFs of ${\hat{X}}_{1}$, ${\hat{X}}_{2}$, … ${\hat{X}}_{N}$ in sequence. The MTSFE architecture is shown in Figure 4.

The designed multi-scale temporal convolutions serve as the initial step in the MTSFE pipeline. A group of multi-scale 2D convolutions (Conv2Ds) with kernel sizes (1,f_s_/2), (1,f_s_/4), (1,f_s_/8), and (1,f_s_/16), where f_s_ is the sampling frequency, is employed to extract features at different temporal resolutions. The temporal Conv2Ds can capture both short-term variations and long-term trends along the time dimension of EEG. After the temporal convolutional operation, the spatial Depthwise 2D convolution (DWConv2D) layers are attached to extract inter-channel (EEG channel) spatial correlations. The kernel sizes of all spatial DWConv2Ds are set to (C,1). Furthermore, the extracted feature maps are processed dual-branch pooling operations, i.e., average pooling (AvgPool) and variance pooling (VarPool), and the kernel sizes are all set to (1,4) with a stride of 20. Average pooling emphasizes preserving global temporal information, and variance pooling highlights variations in feature distributions. We integrated them to enhance the feature representation ability of MTSFE and prevent the loss of key features. Next, the output feature maps by AvgPool ${\overset{⌢}{F}}_{n}={\left\{{\overset{⌢}{f}}_{n,t}\right\}}_{t=1}^{T_{n}}$ and VarPool ${\tilde{F}}_{n}={\left\{{\tilde{f}}_{n,t}\right\}}_{t=1}^{T_{n}}$ are fed to *D* MHSA blocks separately to capture the temporal and spatial dependencies. Taking the AvgPool branch as an example, each row of the input features ${\overset{⌢}{F}}_{n}$ is considered a token and fed into MHSA. The output $O_{n}$ of MHA can be expressed as

(1) $$O_{n}^{\mathrm{NHA}}={\overset{⌢}{F}}_{n}+\mathrm{MHA}\left(\mathrm{LN}\left({\overset{⌢}{F}}_{n}\right)\right),$$

where LN represents the layer normalization operation. Specifically, for the *h*th head of a self-attention layer, query (Q), key (K), and value (V) matrices can be calculated via linear transformations, i.e.,

(2) $$\begin{array}{ccc} Q_{h}={\overset{⌢}{F}}_{n}\cdot W_{Q_{h}}, & K_{h}={\overset{⌢}{F}}_{n}\cdot W_{K_{h}}, & V_{h}={\overset{⌢}{F}}_{n}\cdot W_{V_{h}} \end{array},$$

where $W_{Q_{h}}$, $W_{K_{h}}$, and $W_{V_{h}}$ are trained projection weighting matrixes; then, the self-attention of the *h*th head is

(3) $${head}_{h}=\mathrm{Attention}\left(Q_{h},K_{h},V_{h}\right)=\mathrm{Softmax}\left(\frac{Q_{h}\cdot K_{h}^{T}}{\sqrt{d_{h}}}\right)V_{h},$$

where $d_{h}$ is the dimension of the projection space. Next, multiple self-attention heads are concatenated and then linear mapping performed as follows:

(4) $$\mathrm{MHA}\left(\mathrm{LN}\left({\overset{⌢}{F}}_{n}\right)\right)=\mathrm{Concat}\left({head}_{1},{head}_{2},\ldots ,{head}_{h}\right)W_{h},$$

where $W_{h}$ is the mapping weight matrix that is used to map the output of the multiple self-attention heads to the feature dimensions of the input. In the end layer of MHSA, a multilayer perceptron is introduced to further map the intermediate features nonlinearly. The output feature maps ${\tilde{F}}_{n}$ by VarPool also carry out a similar computation.

Following the MHSA blocks, the output from the AvgPool branch and the VarPool branch are concatenated in the depth direction, and then fed into a convolutional encoder, including a 2D convolutional layer, with a kernel size of (1,2) and the number of filters is $\gamma_{Enc}$, which performs feature integration and prepares the feature representation for classification. After a flatten layer, the extracted TSFs are obtained and output. In particular, in the initial training phase, all blocks participate in the training, while in the incremental learning phases, only the convolutional encoder is fine-tuned, and other blocks are frozen to retain memory for old MI tasks.

#### 2.3.3. Self-Supervised Task Generalization

To improve the generalization ability of $F_{\theta }$ for unseen MI tasks, a self-supervised learning strategy is introduced into $F_{\theta }$ in the initial training phase for constraining the parameter update of the feature extractor with the classification loss. Unlike existing TSF extraction models, to improve the generalizability of $F_{\theta }$ on unseen tasks, we consider the label information while increasing the diversity of the input data during the model training.

Specifically, a projection header $\rho_{p}$ and a classifier header $\rho_{c}$ are attached to the end of $F_{\theta }$ for assisting self-supervised learning. Both of these are personalized to each MI task; in other words, each new MI task needs to retrain a new $\rho_{p}$ and a new $\rho_{c}$. The semantic features ${\hat{Z}}_{1}$ and the predicted distribution ${\hat{P}}_{1}$ of augmented data ${\hat{X}}_{1}$ from the 1st MI task in $\rho_{p}$ and $\rho_{c}$ are obtained by ${\hat{Z}}_{1}=\rho_{p}\circ F_{\theta }\left({\hat{X}}_{1};\theta \right)$ and ${\hat{P}}_{1}=\rho_{c}\circ F_{\theta }\left({\hat{X}}_{1};\theta \right)$, respectively, and θ represents the parameters of $F_{\theta }$. Then, a cross-entropy loss is introduced to measure the difference between ${\hat{P}}_{1}$ with the true distribution ${\hat{Y}}_{1}$. The loss function is given as follows:

(5) $$L_{SSCE}=\sum_{l=1}^{L_{1}}\mathrm{CE}\left({\hat{y}}_{1,l},{\hat{p}}_{1,l}\right),$$

where L is the number of categories included in the initial training, $\mathrm{CE}\left(\cdot \right)$ stands for the cross-entropy loss function, ${\hat{Y}}_{1}={\left\{{\hat{y}}_{1,l}\right\}}_{l=1}^{L}$ and ${\hat{P}}_{1}={\left\{{\hat{p}}_{1,l}\right\}}_{l=1}^{L}$.

In addition, non-augmented EEG recording is separately fed into the same $F_{\theta }$, which forms another non-augmented branch, and the semantic features are computed via $Z_{1}=\rho_{p}\circ F_{\theta }\left(X_{1};\theta \right)$. To improve the stability and computational efficiency of the network, the non-augmented branch is set as gradient-stopping, making the feature distribution of ${\hat{Z}}_{1}$ align to $Z_{1}$ during each iterative update. Thereby, the self-supervised task generalization loss $L_{SSFG}$ can be denoted as

(6) $$L_{SSFG}=1-\frac{1}{L_{1}}\sum_{l=1}^{L_{1}}\mathrm{Cos}\mathrm{Sim}\left({\hat{Z}}_{1,l},\mathrm{Stopgrad}\left(Z_{1,l}\right)\right),$$

where $\mathrm{Cos}\mathrm{Sim}\left(\cdot \right)$ means the cosine similarity computation, i.e.,

(7) $$\mathrm{Cos}\mathrm{Sim}\left({\hat{Z}}_{1,l},\mathrm{stopgrad}\left(Z_{1,l}\right)\right)=\frac{{\hat{Z}}_{1,l}\cdot \mathrm{stopgrad}\left(Z_{1,l}\right)}{\left\|{\hat{Z}}_{1,l}\right\|\left\|\mathrm{stopgrad}\left(Z_{1,l}\right)\right\|}.$$

The self-supervised learning loss $L_{SS}$ can be derived from the task-related cross-entropy loss and the self-supervised task generalization losses as follows:

(8) $$L_{SS}=L_{SSCE}+\lambda_{1}L_{SSFG},$$

where $\lambda_{1}$ is the balance coefficient. In our work, $\lambda_{1}$ is set to 0.5 to enhance guidance on self-supervised learning by the task labels.

#### 2.3.4. Prototype-Guided Generative Replay Module

In the IL phase, to combat the catastrophic forgetting of old knowledge and to protect patients’ data privacy, we consider developing a generative replay model to generate representative features of old tasks and combining them with the new task features to update the convolutional encoder and a unified classifier $G_{W}$. Inspired by conditional diffusion, we retain class prototypes of old tasks and use these prototypes with their real labels as generative conditions, which are replayed in the IL phase, to guide the parameters of DM updating towards the direction of generating features similar to the prototypes. Our work realizes the memory reconstruction of the old task knowledge without relying on the original data, while reducing the storage cost and ensuring consistency of the generated features with the original data.

Generation of task prototypes. The task prototypes ${\left\{\mu_{n}\right\}}_{n=1}^{N}$ describe the key characteristics of the different MI. For the *n*th task; $\mu_{n}$ can be generated by calculating the mean of the eigenvectors, i.e.,

(9) $$\mu_{n}\left({\hat{y}}_{n,t}\right):=\frac{1}{T_{n}}\sum_{t=1}^{T_{n}}F_{\theta }\left({\hat{X}}_{n,t};\theta \right).$$

Then, the task prototypes $\mu_{n}\left({\hat{y}}_{n,t}\right)$ are integrated into the set of seen prototypes $U_{n-1}=\left\{\mu_{1},\mu_{2},\ldots ,\mu_{n-1}\right\}$, denoted as $U_{n}=\left\{\mu_{1},\mu_{2},\ldots ,\mu_{n}\right\}$.

Forward diffusion process. Given a TSF of the newly added *n*th task ${\hat{f}}_{n,t}=F_{\theta }\left({\hat{X}}_{n,t};\theta \right)$, the forward diffusion is designed to gradually add the Gaussian noise to ${\hat{f}}_{n,t}$. After a predefined diffusion process of *K* discrete time steps, it is transformed into an isotropic Gaussian distribution. Specifically, forward diffusion from the initial time step to the 1st time step can be represented as follows:

(10) $$q\left({\hat{f}}_{n,t}^{1}\left|{\hat{f}}_{n,t}^{0}\right.\right):=N\left({\hat{f}}_{n,t}^{1};\sqrt{\left(1-\beta_{0}\right)}{\hat{f}}_{n,t}^{0},\beta_{0}I\right)$$

and

(11) $${\hat{f}}_{n,t}^{1}=\sqrt{\left(1-\beta_{0}\right)}{\hat{f}}_{n,t}^{0}+\sqrt{\beta_{0}}\eta_{0},$$

where $\beta_{0}I$ describes the noise variance schedule to predefine the noise magnitude, clear TSF ${\hat{f}}_{n,t}^{0}∼q\left({\hat{f}}_{n,t}^{0}\left|{\hat{y}}_{n,t}\right.\right)$ denotes the class-conditional data distribution, and ${\hat{y}}_{n,t}$ is the task label. According to Equation (10), the forward diffusion process from a temporal-spatial feature at the first time step ${\hat{f}}_{n,t}^{0}$ to an isotropic Gaussian distribution at the *K*th time step ${\hat{f}}_{n,t}^{K}∼N\left({\hat{f}}_{n,t}^{K},0,I\right)$ is given by

(12) $$Q\left({\hat{f}}_{n,t}^{1:K}\left|{\hat{f}}_{n,t}^{0}\right.,{\hat{y}}_{n,t}\right):=\prod_{k=1}^{K}q\left({\hat{f}}_{n,t}^{k}\left|{\hat{f}}_{n,t}^{k-1}\right.,{\hat{y}}_{n,t}\right).$$

**Figure 5:**
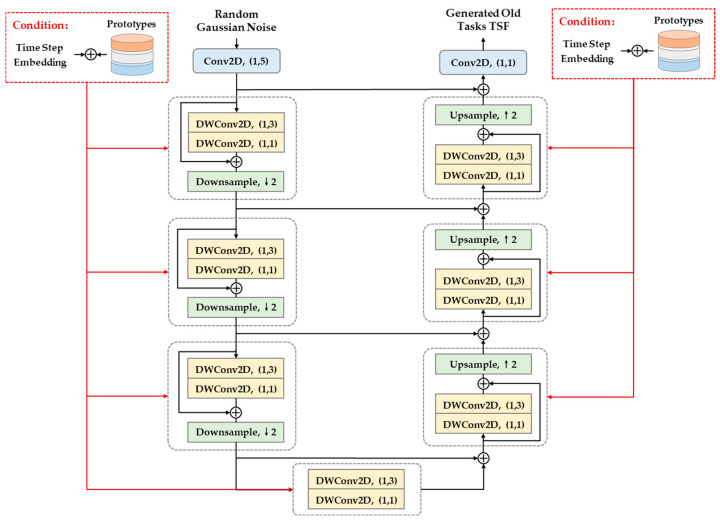
Architecture of LU-Net. Time step embedding with prototypes is employed as a diffusion condition of LU-Net to constrain the generation of old MI task TSF, “↑” represents the upsampling operation, “↓” represents the downsampling operation.

Reverse denoising process. A lightweight U-Net (LU-Net) as a denoising block is introduced to reconstruct the TSFs of old tasks from isotropic Gaussian distributions. In contrast to the conventional U-Net, LU-Net replaces regular convolutions with Depthwise separable convolutions except for the initial and ending layers. Additionally, jump concatenating is reset to addition for feature reuse and dimension reduction. The LU-Net architecture and implementation details are shown in Figure 5; the parameterized LU-Net by α is denoted as $E_{\alpha }$. The reverse denoising process can be represented as follows:

(13) $$a_{\alpha }\left({\hat{z}}_{n,t}^{K-1}\left|{\hat{f}}_{n,t}^{K}\right.,U_{n}({\hat{y}}_{n,t}),{\hat{y}}_{n,t}\right):=N\left({\hat{z}}_{n,t}^{K-1};E_{\alpha }\left({\hat{f}}_{n,t}^{K},K,U_{n}({\hat{y}}_{n,t}),{\hat{y}}_{n,t}\right),{\hat{y}}_{n,t}\right),$$

where the *n*th task prototype $U_{n}({\hat{y}}_{n,t})$ and real label ${\hat{y}}_{n,t}$ are regarded as additional constraint conditions to guide LU-Net updates. Similarly, the reverse denoising process for generating feature ${\hat{z}}_{n,t}^{0}$ from a Gaussian distribution over *K* time steps is accumulated as

(14) $$A_{\alpha }\left({\hat{z}}_{n,t}^{0:K}\left|{\hat{y}}_{n,t}\right.,U_{n}({\hat{y}}_{n,t})\right):=a_{\alpha }\left({\hat{z}}_{n,t}^{K}\left|{\hat{y}}_{n,t}\right.,U_{n}({\hat{y}}_{n,t})\right)\prod_{k=1}^{K}a_{\alpha }\left({\hat{z}}_{n,t}^{k}\left|{\hat{z}}_{n,t}^{k-1}\right.,U_{n}({\hat{y}}_{n,t}),{\hat{y}}_{n,t}\right).$$

Diffusion training of PGGR. To ensure high similarity between the TSFs generated from noise and the real TSFs, the Kullback–Leibler divergence (KL) is minimized to optimize $E_{\alpha }$, which is used to predict the added noise on the forward diffusion process, specifically,

(15) $$\min:J_{LU-Net}=E_{\eta ,{\hat{f}}_{n,t}^{0},K,{\hat{y}}_{n,t}}\left[{\left\|\eta -E_{\alpha }\left({\hat{f}}_{n,t}^{0},K,U_{n}({\hat{y}}_{n,t}),{\hat{y}}_{n,t}\right)\right\|}_{2}^{2}\right].$$

The updated PGGR by the *n*th task is saved and then used to generate the TSFs for all the first *n* tasks ${\hat{Z}}_{1:n}=\left[{\hat{Z}}_{1},{\hat{Z}}_{2},\ldots {\hat{Z}}_{n}\right]$ in the (*n* + 1)th task learning phase.

#### 2.3.5. Training Process

In the initial training phase, an MTSFE block $F_{\theta }$ and a unified classifier $G_{W}$ are updated by the initial MI task, which consists of at least two MI tasks. The total loss function of the initial training is composed of a supervised cross-entropy loss and a self-supervised learning loss as follows:

(16) $$L_{Total}=L_{CE}+\lambda_{2}L_{SSCE},$$

where $L_{CE}=\sum_{l=1}^{L_{1}}\mathrm{CE}\left({\hat{y}}_{1,l},{\hat{g}}_{1,l}\right)$, the classifier output ${\hat{g}}_{1,l}$ is the prediction probabilities of the MI task categories for the *l*th sampling trial, and $\lambda_{2}$ is the penalty factor.

In the incremental training phases, to retain the memory for the old knowledge learned, the multi-scale temporal-spatial convolutions, dual pooling layers, and multiple MHSA in $F_{\theta }$ are frozen. In particular, the attached projection header $\rho_{p}$ and classification header $\rho_{c}$ are personalized for each incremental MI task. Meanwhile, the convolutional encoder is continuously fine-tuned and the classifier $G_{W}$ is updated with newly incorporated EEG data chunks $T_{2}$,…,$T_{N}$.

## 3. Experimental Results

### 3.1. Experimental Settings

According to the dataset size, we designed various ways to divide the training and test sets. For the ADL-MI dataset, the first four sessions are used as the training set, and the last two sessions are used as the test set, with a ratio of 2:1. For the IV-2a dataset, the first session is used as the training set, and the second session as the test set, with a data size ratio of 1:1. In experiments conducted on the same dataset, the dataset division ratio remains consistent.

In the default parameter configuration of the proposed GD-TIL, the temporal segments *S_n_* are set to 8 for data augmentation. In MTSFE, the number of temporal and spatial filters for all scales is set to 8 and 2, respectively. The depth of the MHSA block *D* and the number of attentional heads *h* are determined as 4 and 8, respectively. These settings are the same as the parameter settings of many reported papers [40]. During training the MTSFE, a maximum of 2000 epochs and a batch size of 64 are set to minimize the total loss function. A default Adam optimizer is applied to optimize the model, with a learning rate of 0.01. The balance coefficient $\lambda_{1}$ in Equation (8) is empirically given as 0.5. The implementation details of PGGR have been given in Figure 5, where the number and dimensionality of the generated TSFs are the same as new MI tasks. All experiments are conducted on an HP workstation (Shanghai HP Co., Ltd., Shanghai, China) equipped with an 80 GHz Intel^®^ Core™ i7 processor, 80 GB RAM, and an NVIDIA T1000 GPU, and NVIDIA GeForce RTX 3090 GPU, CUDA 10.2 and cuDNN 7605. The model simulation uses Python 3.9 and Pytorch 1.10 in Spyder 5.1.5.

### 3.2. Evaluation Metrics

The average decoding accuracy (*Acc*.) is utilized to measure the decoding ability of the model for each task, including the initial and incremental tasks, and it can be expressed as the following equation:

(17) $$Acc.=\frac{1}{N}\sum_{n}^{N}acc_{N,n},$$

where $acc_{N,n}$ represents the decoding accuracy of the model for the *n*th task after being updated by the *N*th task. In addition, Cohen’s kappa coefficient (*Kappa*) is also introduced to evaluate the consistency of the classification results; it can be computed based on the confusion matrix. *Acc*. provides an intuitive measure of a model’s correctness in overall classification tasks. *Kappa* further quantifies the reliability of classification performance, particularly in scenarios with imbalanced class distributions, serving as an indicator of the agreement between the model’s predictions and the actual labels.

The forward transfer rate (*FTR*) [41,42] is introduced to evaluate the plasticity of an IL model, in other words, the model’s ability to learn unseen tasks continuously. *FTR* defines the change rate in the decoding accuracies of the model for a given task when it is learned jointly and as the *n*th incremental task. *FTR* can be calculated as follows:

(18) $$FTR=\frac{1}{N-1}\sum_{n=2}^{N}\frac{acc_{n}^{**}-acc_{n,n}}{acc_{n}^{**}},$$

where $acc_{n}^{**}$ means the decoding accuracy of the model for the *n*th joint learning task. Generally, a smaller *FTR* indicates better plasticity of the IL model.

The backward forgetting rate (*BFR*) measures the stability of the IL model for decoding seen tasks. *BFR* is the degree of decay in the decoding accuracy between when a task is first learned and after learned on the final task, i.e.,

(19) $$BFR=\frac{1}{N-1}\sum_{n=1}^{N-1}\frac{acc_{n}^{*}-acc_{N,n}}{acc_{n}^{*}},$$

where $acc_{n}^{*}$ stands for the decoding accuracy when the model first learns the *n*th task. A low *BFR* suggests that the model memorizes old knowledge well, while a high *BFR* reflects the phenomenon of “catastrophic forgetting”.

### 3.3. Decoding Performance Evaluation

#### 3.3.1. Evaluation on the ADL-MI Dataset

The decoding performance of GD-TIL is first evaluated on incremental MI task pairs. The decoding results for each subject on the initial task (single MI pair) and the incremental task (two MI pairs) are listed in Table 1. GD-TIL shows superior results in decoding both the initial and incremental tasks. For the initial task of binary classification, the average *Acc.* is 87.04% with an *Std*. of 6.7 and the *Kappa* coefficient is 0.7409, indicating that GD-TIL can accurately capture the differences in TSF between tasks and make decisions while showing high stability in predicting different categories. Furthermore, when a new MI pair is incorporated, the average *Acc*. for the four-classification task is 80.20% with an *Std*. of 7.88 and the *Kappa* coefficient is 0.7359, which show our work’s strong self-updating and continuous learning ability, as the classification accuracy only decays by 6.84% despite doubling of the number of categories. Although the decoding results varied across subjects due to inter-individual differences, i.e., A3 performs the best with 93.62% and 89.05% *Acc*. for single and incremental MI pairs, respectively, whilst A5 performs the lowest with 75.60% and 69.47%, respectively; the low *Std*. implies that GD-TIL has excellent generalizability for different subjects.

#### 3.3.2. Evaluation on the IV-2a Dataset

Furthermore, GD-TIL is evaluated on the IV-2a dataset with multiple incremental MI tasks, and the decoding results are shown in Table 2. As new MI tasks are gradually incorporated, the number of categories is subsequently increased, resulting in more difficult classification and a decrease in the decoding performance of GD-TIL. The average *Acc*. decreases from 90.11% in the initial learning stage to 85.60% in the incremental learning phase with three MI tasks and to 81.32% for the four MI tasks, while the *Kappa* coefficient decreases from 0.8021 to 0.7509. Despite the decrease in performance, its magnitude is small, which implies that the model is able to learn new tasks efficiently and has a strong incremental learning ability even in the face of multiple unknown new tasks, while maintaining the performance of old tasks better. In addition, regarding the performance in coping with subject variability, the *Acc*. of B1 in the incremental phase with four MI tasks is 89.66%, whereas B2 is 62.86%, but the *Std*. of only 8.65 signifies that the overall performance of GD-TIL is still robust, and the excellent inter-subject generalization ability is proved once again. Overall, GD-TIL can effectively adapt to new tasks and maintains high decoding performance in multi-task incremental learning, demonstrating its excellent adaptivity and robustness.

### 3.4. Plasticity Evaluation

#### 3.4.1. Evaluation on the ADL-MI Dataset

**Figure 6:**
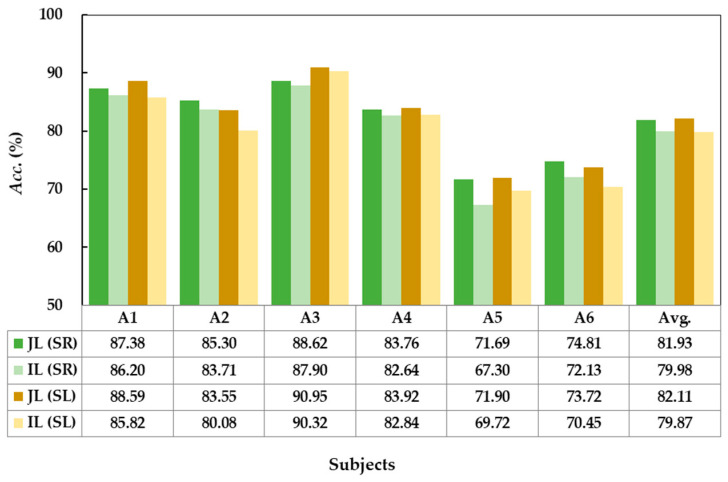
Comparison of decoding accuracy between joint learning (JL) and incremental learning (IL) of GD-TIL for the added arm sideways raising (SR) and lowering (SL), respectively.

To evaluate the learning ability of GD-TIL for unknown tasks, on the ADL-MI dataset with an incremental MI pair, the *Acc*. of incremental learning (IL) for the added MI pair, including arm sideways raising (SR) and lowering (SL), is compared with its *Acc.* in joint learning (JL) in Figure 6. The average *Acc.* of the SR task is 82.79% in IL, which is only 1.12% lower than the 83.91% in JL, and the average *Acc.* of the SL task is 79.87% in IL, which is 2.24% lower than the 82.11% in JL. Across subjects, IL achieves accuracies comparable to JL for both SR and SL. Comparative results show that GD-TIL can effectively adapt to new tasks and maintain a high level of performance consistency when learning new tasks incrementally.

To further analyze the plasticity of the proposed GD-TIL, the *FTRs* of each subject with their average value are listed in Table 3; a lower *FTR* means that the incremental learning ability of the model is better. For the SR task, the *FTRs* of A3 and A4 are 0.69% and 1.29%, respectively, which are significantly lower than the other subjects, and for the SL task, the *FTRs* of A3 and A4 are 0.75% and 1.31%, which are also at the lowest level, reflecting the superior IL ability of GD-TIL on these subjects. Overall, the average *FTRs* of GD-TIL on SR and SL are 2.79% and 2.65%, respectively; both of them are kept at a low level (less than 5%), which indicates that the model is able to effectively adapt to the new MI pair during the IL process and demonstrates excellent plasticity and cross-task adaptation ability.

#### 3.4.2. Evaluation on the IV-2a Dataset

**Figure 7:**
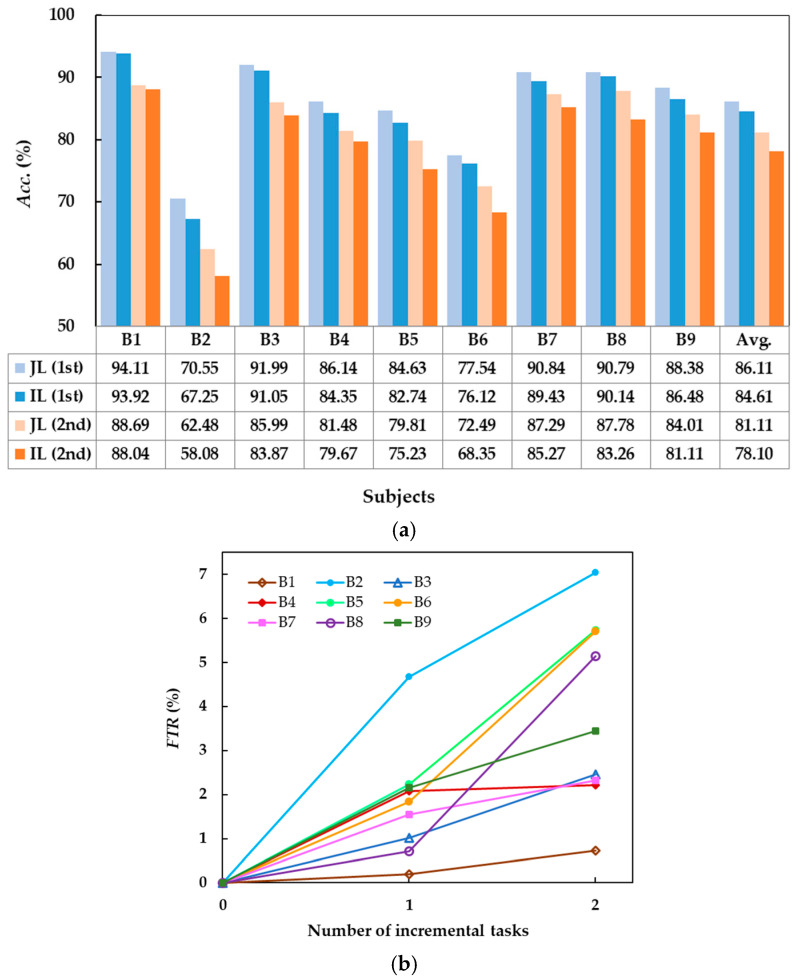
Comparison of joint learning (JL) and incremental learning (IL) of GD-TIL for the first (1st) and second (2nd) added MI tasks. (**a**) Average decoding accuracies; (**b**) Forward transfer rate.

The decoding accuracies of GD-TIL for JL and IL on the two incremental MI tasks are compared in Figure 7a. In the 1st incremental task, the average *Acc*. of JL reaches 86.61%, and it stays at 81.11% for the IL task, with a gap of only 3.5%. In the 2nd incremental task, the average accuracies of JL and IL are 81.11% and 78.10%, respectively. Despite the slightly larger gap, the performance of GD-TIL for IL still shows strong learning ability; especially for subjects B7 and B8, the *Acc*. of IL is even close to that of JL. The difference between the results obtained by GD-TIL in learning a new MI task by self-updating and the results obtained by the batch mode is small. The above comparison results demonstrate that the learning ability and adaptability of our method to unknown MI tasks are superior even on multiple incremental tasks.

Furthermore, Figure 7b gives the *FTR* of GD-TIL on the 1st and 2nd incremental tasks for different subjects. Although some subjects (e.g., B2 and B6) show a faster increase in FTR on the 2nd task than on the 1st task, some subjects (e.g., B3 and B8) show a decreasing FTR growth rate trend. In addition, most subjects (e.g., B1, B7, and B9) exhibit relatively slow FTR growth, which is consistently low, with an average lower than 5%. The experimental results again demonstrate the excellent plasticity of GD-TIL to unknown knowledge.

### 3.5. Stability Evaluation

#### 3.5.1. Evaluation on the ADL-MI Dataset

To evaluate the memory of GD-TIL for old tasks during incremental learning, i.e., the plasticity of the model, the *BFR* for old FR and FL tasks and their average *BFRs* are introduced in Table 4. Most of the subjects have low *BFRs*; in particular*,* for subjects A3 and A4, average *BFRs* of 4.95% and 4.68% are observed, respectively. Possibly influenced by the quality of EEG, only A6 show a significantly higher *BFR* with a mean value of 14.93%. From the overall results, the average *BFRs* of GD-TIL is 7.6%, where the average *BFRs* of FR and FL were 7.65% and 7.55%, respectively. The anomaly in A6 may suggest the need to further investigate the model’s ability to adapt to different individual difference in future work to further optimize the overall performance. The experimental results suggest that GD-TIL has better stability for remembering old knowledge; it also means that our IL strategy can effectively alleviate the catastrophic forgetting.

#### 3.5.2. Evaluation on the IV-2a Dataset

**Figure 8:**
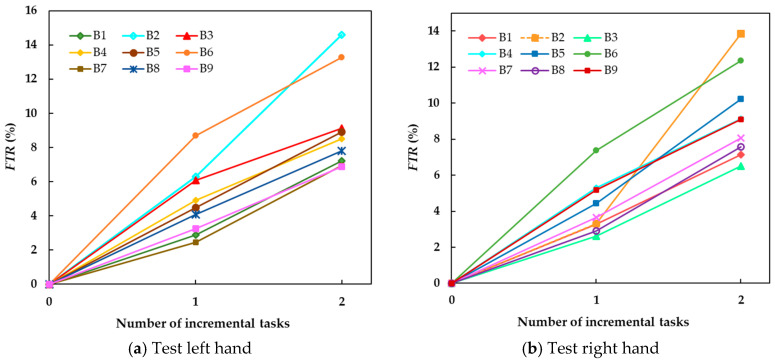

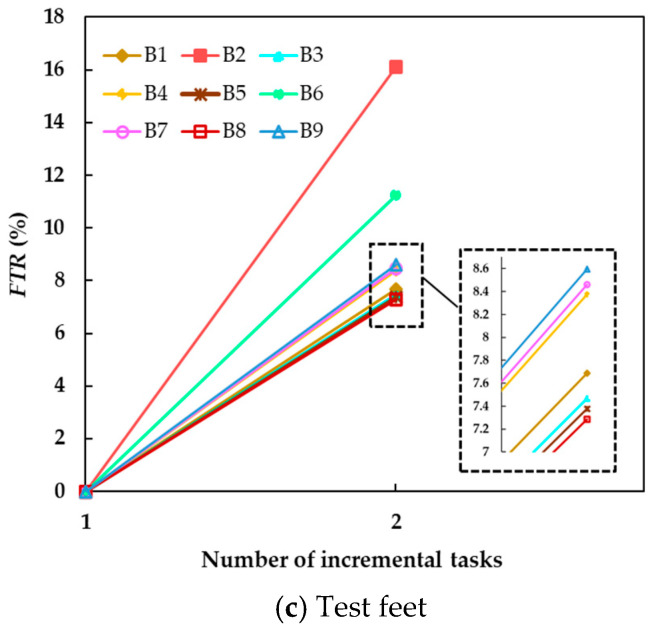
Backward forgetting rates for old tasks after GD-TIL learns one or more new tasks, where (**a**,**b**) show the updated GD-TIL test results for the initial task (1st and 2nd), including left-hand and right-hand MI tasks, respectively, and (**c**) shows the test results for the feet (3rd) MI task.

Figure 8 gives the *BFRs* for old tasks after GD-TIL learns one or more new MI tasks. The *BFRs* for the initial two MI tasks (left hand and right hand) are shown in Figure 8a,b, and the *BFR* for the 1st incremental MI task (feet) after learning the 2nd incremental MI task (tough) is shown in Figure 8c. As the number of tasks increases, the forgetting of old tasks resulting from increase in categories is inevitable. However, the *BFRs* remain at low values for most of the subjects, indicating that the model performs without suffering from catastrophic forgetting. In particular, the results in Figure 8c show remembering of the 1st MI task after learning a few incremental MI tasks. Overall, the experimental results demonstrate the model’s wonderful old knowledge stability and resistance to forgetting in response to multi-task incremental learning.

### 3.6. Ablation Study

*Impact of DA and SSTG on decoding performance.* Table 5 displays the joint training results of the ablation experiments for the impact of DA and SSTG on decoding performance, including binary and multi-class MI tasks. MTSFE with DA and SSTG performs the best, significantly improving *Acc*. and *Kappa* in all tasks. Only DA shows a relatively limited improvement in the experimental results, while the continuing addition of SSTG significantly improves the decoding performance, especially for the results of multi-class MI tasks. Overall, a combination of DA, and SSTG with MTSFE is optimal for temporal-spatial feature representation and classification.

**Figure 9:**
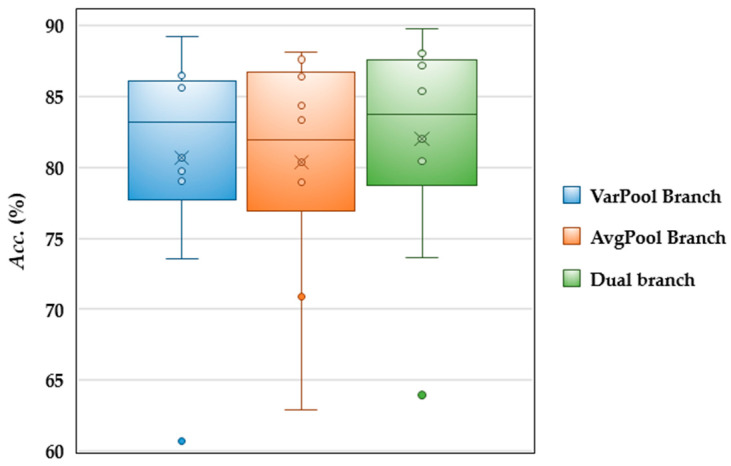
Comparison of average decoding accuracies for different pooling operations.

*Comparison of different pooling operations.* The *Accs*. of different pooling operations are compared in Figure 9. The AvgPool and VarPool branches have their own advantages in improving the decoding performance, but the dual branch method combines the features of both and shows the best decoding performance. Specifically, the median of the dual branch layer is higher than the single pooling layer, while its mean is also at a high level, and the number of outliers is the least, which further indicates the greater consistency of integrating the dual branch layer. All comparison results indicate that the dual branch pooling layer is able to capture global and local features more effectively.

**Figure 10:**
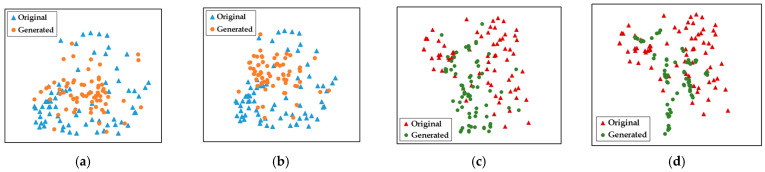
Visualization of TSFs distribution by the t-SNE method. (**a**) LH (after adding the 1st task), (**b**) LH (after adding the 2nd task), (**c**) RH (after adding the 1st task), (**d**) RH (after adding the 2nd task).

*Contribution of PGGR to IL performance.* The visualization results of TSFs by t-SNE are shown in Figure 10, including the distribution changes of left-handed MI (LH) and right-handed MI (RH) during incremental learning. According to the feature distribution, we can observe that the generated TSFs have significant overlap with the original TSFs in the distribution space. After the introduction of multiple new MI tasks, the generated TSFs can still accurately reflect the distribution characteristics of the original TSFs without significant distortion. The above results show that the proposed PGGR has high fidelity and robustness in the generation process and can effectively support the smooth implementation of IL MI tasks.

### 3.7. Parameter Sensitivity Analysis

**Figure 11:**
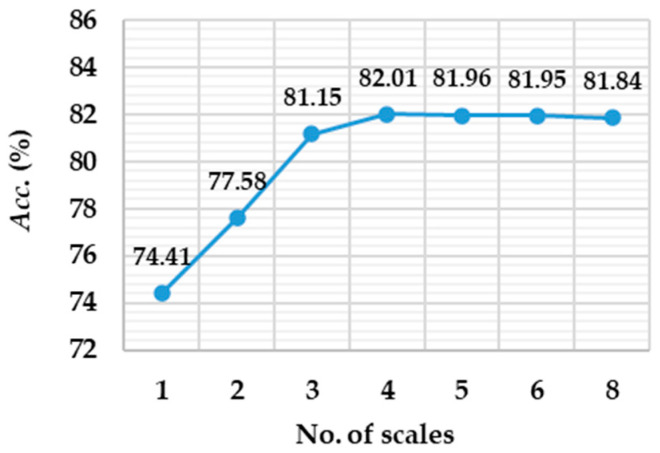
Comparison of average decoding accuracy for different temporal and spatial convolution scale settings.

*Temporal and spatial convolution scales.* According to Figure 11, in the case of joint training, the *Acc*. of the proposed method shows an obvious trend of rapid increase followed by gradual stabilization with increase in the number of scales. For the number of scales from 1 to 4, the decoding performance is significantly improved, which means multiple feature extraction branches can effectively capture the temporal-spatial information. However, when the number of scales exceeds 4, the improvement in *Acc*. tends to saturation, reflecting that further increasing the number of scales gradually reduces the marginal gain in performance. Considering the improvement in decoding performance and the increase in computational cost, a scale number of 4 is selected to balance performance and efficiency.

*Impact of the penalty factor on IL performance.* Table 6 presents the impact of varying penalty factors λ_2_ on IL performance. The comparison results show that the *Acc*. achieves the best performance when λ_2_ is 0.8. Although *FTR* and *BFR* are not at the minimum values, they differed from the optimal value by only 0.03 and 0.11, respectively. The *FTR* and *BFR* show gradual decreases with increase in λ_2_, and this trend suggests that the introduced self-supervised learning constraints contribute to balancing the plasticity and stability of the proposed method during the IL process, thereby improving the overall performance of GD-TIL.

### 3.8. Decoding Performance Comparison

The decoding performance of our method DG-TIL is compared with related work on the IV-2a dataset. The *Acc*. and average results for each subject are shown in Table 7. Gao et al. proposed a parallel-structured feature fusion network and evaluated it on the four-class MI task [5]. Other compared related works include the multi-branch networks EEG-FMCNN [11] and TWSB [27], the advanced TSF extraction methods GSAN [43] and IFBCLNet [44], and M-FANet [45], which introduced self-attention mechanisms to improve decoding performance. What is more, BLS [46], ASiBLS [42], and SIBLS [41] were the only reported IL methods for MI-EEG. According to Table 6, our method performs the best among all methods with an average *Acc*. of 81.32%, which is superior to the related methods being compared. In particular, compared to ASiBLS and SiBLS, DG-TIL improves the average *Acc*. by 1.43% and 1.7%, respectively, showing a significant advantage in incremental learning scenarios. The comparison results indicate that DG-TIL has stronger capabilities in model updating and old knowledge retention. In addition, DG-TIL also controls the *Std.* of individuals in a low range. DG-TIL outperforms existing methods in decoding accuracy and stability. It shows superiority in multi-task BCI study and further combines the effectiveness of multi-scale TSF extraction and MI task incremental learning.

## 4. Discussion

In this paper, the proposed GD-TIL achieves a balance between plasticity for new MI tasks and stability for retaining old tasks by integrating data augmentation, multi-scale feature extraction, self-supervised task generalization, and generative replay strategies. Moreover, on a self-collected ADL-MI dataset and a public dataset, GD-TIL achieves continuous decoding accuracies of 80.20% and 81.32%, respectively. These results not only outperform state-of-the-art incremental learning methods but also underscore the potential of MI-based BCIs combined with generative AI for advancing neurorehabilitation.

The proposed GD-TIL exhibits robust classification performance and strong incremental learning capabilities across subjects and motor imagery (MI) tasks, as demonstrated by evaluations on the ADL-MI and IV-2a datasets. Table 1 shows that GD-TIL achieves an average accuracy of 87.04% on a single MI pair and 80.20% on the incremental task of two MI pairs on the ADL-MI dataset. Although there are differences in individual performance, A3 had the highest accuracy of 89.05% on the multiple MI tasks and A5 had the lowest of 69.47%. The lower *Std*. of 7.88 suggests excellent generalization between subjects. Similarly, on the IV-2a dataset, Table 2 shows that GD-TIL maintains a high performance, decreasing from 90.11% on the binary task to 81.32% on the four-category incremental task. The results of the decoding performance evaluation highlight the GD-TIL’s adaptability to different MI tasks while ensuring inter-subject performance consistency.

Furthermore, GD-TIL exhibits desirable plasticity and stability, which are crucial for task incremental learning. On the ADL-MI dataset, Table 3 demonstrates that the FTRs are 2.79% and 2.65% for the SR and SL MI tasks, respectively. On the IV-2a dataset, the FTRs remain consistently low across subjects, as shown in Figure 7a, even with the addition of up to four MI tasks. Regarding stability, Table 4 shows an average BFR of 7.6% on the ADL-MI dataset, with A6 exhibiting the highest BFR at 14.93%. On the IV-2a dataset, Figure 8 illustrates that the BFRs for old MI tasks remain low across subjects, even as new tasks are incrementally introduced. The low FTR indicates that GD-TIL can effectively acquire new knowledge with minimal interference with old knowledge, while the low BFR confirms that previously learned knowledge is retained even when new tasks are introduced. The trade-off between plasticity and stability ensures that the GD-TIL is well suited for real-world scenarios that require continuous adaptation, such as neurorehabilitation of stroke patients that require the incorporation of new MI tasks without loss of proficiency on previous MI tasks. Meanwhile, GD-TIL shows stable performance across subjects and task complexity, further demonstrating the strong adaptability of GD-TIL to effectively cope with individual differences in EEG signals as well as inter-task variability.

The experimental results of the ablation study and parameter sensitivity analysis rigorously demonstrate that each component of the proposed GD-TIL plays a crucial role in improving the IL performance. As shown in Table 5, the combination of DA and SSTG significantly improves the decoding performance, especially in multi-class MI tasks, by making TSF more diverse and representative. Figure 9 gives an example of the two-branch pooling structure that effectively captures both global and local features, yielding more robust and consistent decoding accuracy compared to a single pooling method. And PGGR ensures high fidelity in preserving the TSFs of old MI tasks, as evidenced by the t-SNE visualization shown in Figure 10, which illustrates minimal distortion of the feature distribution during incremental learning. Parameter sensitivity analysis further highlights the importance of balancing performance and efficiency. Figure 11 shows optimal results for a temporal convolution scale of 4, while Table 6 shows optimal results for a penalty factor *λ_2_* of 0.8. These components enable GD-TIL to efficiently balance plasticity and stability, which mitigate catastrophic forgetting while maintaining strong generalization and adaptability to new MI tasks.

In addition, the objective of SSTG is to learn robust TSF representations by constructing auxiliary tasks, i.e., MI tasks after DA. To enhance the TSF representation ability of MTSFE and its adaptability to incremental MI tasks, TSFs from the augmented MI-EEG are further mapped and optimized through a projection head $\rho_{p}$ and an additional classification head $\rho_{c}$. $\rho_{p}$ maps TSFs into a high-dimensional space, generating semantic features that improve sample clustering for the same MI tasks and separation for different MI tasks. And $\rho_{c}$ provides direct supervision for the auxiliary MI tasks, while ensuring that the TSFs learned by the classifier $G_{W}$ are more distinguishable. Many reported self-supervised learning methods like SimCLR [47] and BYOL [48] have suggested that removing the projection header or classification header disrupts hierarchical feature learning, so the classification effectiveness of the model in downstream tasks is significantly reduced. This finding implies the irreplaceable role of $\rho_{p}$ and $\rho_{c}$ in optimizing TSF representation and enhancing IL performance.

As a method specifically designed to meet the challenges of BCI scenarios with incremental tasks, the comparison results of the decoding performance of DG-TIL with non-incremental and IL methods highlight the superiority of DG-TIL in both decoding accuracy and IL ability, as shown in Table 7. Compared to state-of-the-art IL methods, such as ASiBLS [42] and SiBLS [41], DG-TIL achieves an obvious improvement in decoding accuracy. This advantage stems from the integration of advanced feature extraction and effective IL strategies. In contrast, ASiBLS and SiBLS face limitations in preserving old knowledge effectively, which impacts their overall performance in multi-task settings. Non-incremental methods, such as GSAN [43], M-FANet [45], and EEG-FMCNN [11], exhibit reasonable decoding performance but lack the adaptability required for IL scenarios. DG-TIL not only surpasses these methods in decoding performance but also demonstrates better stability across subjects, as evidenced by its lower variability in individual results. While GSAN and M-FANet focus primarily on TSF extraction, they struggle to maintain stable performance when new MI tasks are introduced. DG-TIL balances feature adaptability and knowledge retention, making it a more comprehensive and effective solution for decoding incremental MI tasks.

Frankly speaking, our method still has some limitations. Firstly, although the generalization of GD-TIL can be demonstrated by subject-independent experiments, higher computational costs are inevitable when the model is re-trained for each subject. In the next work, we will strive to develop the self-adaptability of the IL models, enabling across-subject inference without repetitive training. Specifically, to reduce computational cost and maintain high decoding performance, we intend to introduce transfer learning techniques to quickly adapt pre-trained models for new subjects and to explore meta-learning methods or task-based fine-tuning strategies for improving model generalization across subjects. The complete resolution of the forgetting issue in IL models is also a goal of our future work. We will focus on improving IL strategies by integrating more robust knowledge retention mechanisms, such as prototype-based memory modules or dynamic task-specific regularization. In addition, in this study, EEG signal segmentation and restructuring are the only DA techniques used in SSTG. The addition of noise or more advanced generative models, such as GANs and other generative models, can also create synthetic data that mimic the underlying distribution of the EEG signals. However, the computational cost and the risk of generating samples inconsistent with the actual signal distribution remain challenges. In the future, we will explore GAN-based methods or hybrid strategies combining multiple approaches to achieve a balance between computational efficiency and model robustness. Moreover, we also plan to further incorporate alpha rhythm-specific processing methods, such as finer frequency banding or direct analysis of neurophysiological features, to enhance the physiological interpretability of the proposed method.

## 5. Conclusions

In this paper, a novel task IL method for EEG-based MI decoding is proposed, i.e., generative diffusion-based task incremental learning (GD-TIL). First, we perform data augmentation to increase EEG diversity by slicing and reorganizing the original signal. Next, the augmented data are fed into a developed MTSFE to extract TSFs. MTSFE integrates multi-scale temporal-spatial convolutions, a dual pooling layer branches along with multiple MHSAs, and a convolutional encoder is attached after the MHSAs for fusing the dual-pooled branch’s global features, which are weighted outputs by the MHSAs. Next, to improve the generalization ability of MTSFE for unknown tasks added subsequently, the SSTG mechanism is introduced and guides MTSFE together with the labeled classification loss. Meanwhile, we designed a PGGR for replaying TSFs of the old task based on a prototype in the IL phases, while the generated TSFs of old tasks with the TSFs of the new task are used to fine-tune the unfrozen convolutional encoder and classifiers, and update the PGGR to prepare for subsequent tasks. Finally, our work is evaluated on a self-collected ADL-MI dataset and a public dataset. Experimental results show that GD-TIL can balance plasticity to new tasks and stability to old tasks well, and its decoding performance outperforms related methods, which include state-of-the-art TSF extraction methods and IL methods. Our work provides a promising approach for developing MI-based BCI in a clinical neurorehabilitation setting, as it combines an efficient feature extraction process with advanced incremental learning strategies, highlighting the potential of using generative AI models in adaptive neurorehabilitation scenarios.

## Figures and Tables

**Table 1:** Decoding results of GD-TIL for incremental MI pairs on the ADL-MI dataset.

Subjects	Single MI Pair		Two MI Pairs (+1) ^1^	
	*Acc*.	*Kappa*	*Acc*.	*Kappa*
A1	93.26	0.8651	85.96	0.8128
A2	89.18	0.7835	82.71	0.7694
A3	93.62	0.8724	89.05	0.8539
A4	85.97	0.7194	82.34	0.7646
A5	75.60	0.5120	69.47	0.5930
A6	84.64	0.6927	71.64	0.6219
Avg.	87.04	0.7409	90.20	0.7359
Std.	6.70	-	7.88	-

^1.^ “+1” stands for adding a task.

**Table 2:** Decoding results of GD-TIL for incremental tasks on the IV-2a dataset.

Subjects	Binary Tasks		Three Tasks (+1) ^1^		Four Tasks (+2)	
	*Acc*.	*Kappa*	*Acc*.	*Kappa*	*Acc*.	*Kappa*
B1	97.12	0.9423	94.05	0.9107	89.66	0.8621
B2	75.01	0.5001	70.02	0.5503	62.86	0.5048
B3	94.64	0.8928	90.70	0.8605	86.70	0.8227
B4	89.77	0.7953	84.90	0.7735	81.68	0.7558
B5	88.17	0.7633	83.74	0.7561	79.34	0.7245
B6	83.47	0.6694	76.55	0.6483	72.59	0.6346
B7	95.50	0.9099	91.53	0.8729	87.55	0.8339
B8	94.15	0.8829	90.61	0.8592	86.28	0.8171
B9	93.15	0.8629	88.31	0.8246	85.20	0.8027
Avg.	90.11	0.8021	85.60	0.7840	81.32	0.7509
Std.	7.07	-	7.86	-	8.65	

^1^ “+1” stands for adding a task.

**Table 3:** Forward transfer rates (%) of GD-TIL for the added arm sideways raising (SR) and lowering (SL).

MI Task	A1	A2	A3	A4	A5	A6	Avg.
SR	3.13	4.15	0.69	1.29	3.03	4.44	2.79
SL	2.24	3.01	0.75	1.31	4.58	4.01	2.65

**Table 4:** Backward forgetting rates (%) of GD-TIL for the old forward raising (FR) and lowering (FL).

MI Task	A1	A2	A3	A4	A5	A6	Avg.
FR	9.09	6.97	4.18	4.33	7.27	14.09	7.65
FL	6.64	5.72	5.71	5.04	6.41	15.77	7.55
avg.	7.87	6.35	4.95	4.68	6.84	14.93	7.60

**Table 5:** Impact of data augmentation (DA) and self-supervised task generalization (SSTG) on decoding performance.

Methods	*Acc.* ± *Std.* (%)			*Kappa*		
	Binary Tasks	Three Tasks	Four Tasks	Binary Tasks	Three Tasks	Four Tasks
MTSFE	87.01 ± 6.91	82.2 ± 7.55	79.43 ± 8.22	0.7693	0.7528	0.7298
DA + MTSFE	87.53 ± 6.21	83.38 ± 7.44	80.53 ± 8.54	0.7725	0.7562	0.7379
DA + MTSFE + SSTG	90.11 + 7.07	86.10 + 7.61	82.01 + 8.40	0.8021	0.7915	0.7602

**Table 6:** Impact of the penalty factor on IL performance.

λ_2_	*Acc.*	*FTR*	*BFR*
0	80.53	4.63	10.66
0.1	80.59	4.15	10.37
0.2	80.71	4.36	10.5
0.4	80.36	4.02	10.21
0.5	81.22	4.07	9.85
0.6	81.27	**3.84**	9.56
0.8	**81.32**	3.87	9.29
1	81.16	3.98	**9.18**

Note: The bold font is the best result.

**Table 7:** Comparison of the decoding performance on the IV-2a dataset.

Methods	B1	B2	B3	B4	B5	B6	B7	B8	B9	Avg.	Std.	*p*-Value
Gao et al. [5]	88.30	72.80	93.70	76.20	60.20	71.10	84.10	96.40	83.70	80.72	11.67	0.4246
EEG-FMCNN [11]	82.29	53.47	93.06	72.22	79.17	71.18	87.85	83.68	86.46	78.82	11.85	0.0985
GSAN [43]	87.60	59.76	90.87	80.38	57.71	51.04	92.01	87.08	87.92	77.15	16.22	0.1283
TWSB [27]	89.30	66.90	89.30	69.30	74.10	60.10	89.40	88.00	85.60	79.11	11.55	0.1638
M-FANet [45]	86.81	75.00	91.67	73.61	76.39	61.46	85.76	75.69	87.15	79.28	9.38	0.2219
IFBCLNet [44]	87.18	58.65	92.67	78.07	70.65	60.46	92.41	82.28	86.74	78.79	12.91	0.1178
BLS [46]	73.86	56.07	66.88	68.26	58.83	59.60	65.10	64.15	60.56	63.70	5.53	<0.01
ASiBLS [42]	85.17	75.83	86.71	73.71	79.2	68.78	82.91	83.2	83.46	79.89	5.98	0.2449
SIBLS [41]	85.40	69.03	87.32	63.31	82.74	72.84	86.28	85.97	83.7	79.62	8.86	0.2417
DG-TIL (Ours)	89.66	62.86	86.70	81.68	79.34	72.59	87.55	86.28	85.20	81.32	8.65	-

## Data Availability

The data presented in this study are available on request from the corresponding author due to the privacy of the subjects.

## Abbreviations

ADL	Activities of Daily Living	DA	Data Augmentation
GD-TIL	Generative Diffusion-Based Task Incremental Learning Method	STG	Self-Supervised Task Generalization
BCI	Brain-Computer Interfaces	GAN	Generative Adversarial Network
MI	Motor Imagery	DM	Diffusion Model
EEG	Electroencephalogram	DDPM	Denoising Diffusion Probabilistic Model
IL	Incremental Learning	MI-AR	Assisted Rehabilitation Platform Oriented to MI-BCI
TSF	Temporal-Spatial Feature	ICA	Independent Component Analysis
MTSFE	Multi-Scale Temporal-Spatial Feature Extractor	EMG	Electromyographic
SSTG	Self-Supervised Task Generalization	MHA	Multi-Head Attention Mechanism
PGGR	Prototype-Guided Generative Replay Module	MHSA	Multi-Head Self-Attention Mechanism
PT	Physical Therapy	Conv2D	2D Convolution
OT	Occupational Therapy	DWConv2D	Depthwise 2D Convolution
FR	Left Arm Forward Raising	AvgPool	Average Pooling
FL	Left Arm Forward Lowering	VarPool	Variance Pooling
SR	Left Arm Sideways Raising	LU-Net	Lightweight U-Net
SL	Left Arm Sideways Lowering	KL	Kullback–Leibler Divergence
LH	Left Hand	Acc.	Average Decoding Accuracy
RH	Right Hand	FTR	Forward Transfer Rate
F	Feet	BFR	Backward Forgetting Rate
T	Tongue	Kappa	Cohen’s Kappa Coefficient
JL	Joint Learning		
